# Skeletal muscle in MuRF1 null mice is not spared in low-gravity conditions, indicating atrophy proceeds by unique mechanisms in space

**DOI:** 10.1038/s41598-019-45821-9

**Published:** 2019-06-28

**Authors:** Samuel M. Cadena, Yunyu Zhang, Jian Fang, Sophie Brachat, Pia Kuss, Elisa Giorgetti, Louis S. Stodieck, Michaela Kneissel, David J. Glass

**Affiliations:** 1 Novartis Institutes for Biomedical Research, 181 Massachusetts Avenue, Cambridge, MA 02139 USA; 2 Novartis Institutes for Biomedical Research, Novartis Campus, 4056 Basel, Switzerland; 3 BioServe Space Technologies, Department of Aerospace Engineering Sciences, University of Colorado, Boulder, CO 80309 USA

**Keywords:** Mechanisms of disease, Medical research

## Abstract

Microgravity exposure is associated with loss of muscle mass and strength. The E3 ubiquitin ligase MuRF1 plays an integral role in degrading the contractile apparatus of skeletal muscle; MuRF1 null (KO) mice have shown protection in ground-based models of muscle atrophy. In contrast, MuRF1 KO mice subjected to 21 days of microgravity on the International Space Station (ISS) were not protected from muscle atrophy. In a time course experiment microgravity-induced muscle loss on the ISS showed MuRF1 gene expression was not upregulated. A comparison of the soleus transcriptome profiles between spaceflight and a publicly available data set for hindlimb suspension, a claimed surrogate model of microgravity, showed only marginal commonalities between the models. These findings demonstrate spaceflight induced atrophy is unique, and that understanding of effects of space requires study situated beyond the Earth’s mesosphere.

## Introduction

Muscle wasting on Earth is seen as a result of many pathological conditions, including cancer cachexia, trauma, sepsis, long-term age-associated diseases such as chronic kidney disease, heart, disease and COPD^1,2^. Muscle atrophy is also observed simply as a result of inactivity, such as when a limb is casted, and as a result of aging, as seen in settings of sarcopenia^1,3^. Loss of skeletal muscle mass results in weakness, reduced mobility and impaired quality of life. Indeed, long‐term muscle wasting is a major risk factor which has been linked to an increase in mortality^4^.

The E3 ubiquitin ligase MuRF1 (Muscle‐specific RING‐FINGER 1) undergoes an increase in acute expression in multiple models of skeletal muscle atrophy, including immobilization, hindlimb suspension (as a model of muscle unloading) glucocorticoid treatment, denervation, and cachexia^1^. MuRF1 knockouts were found to have less skeletal muscle atrophy in all of the mouse atrophy models where it has been studied on Earth^5,6^. For example, MuRF1 KO in skeletal muscle is partially protected from atrophy in denervated or immobilized animals^5^. MuRF1 targets the important sarcomere proteins myosin heavy chain^7^, myosin light chain and other sarcomeric components which have myosin domains^8^ and thus plays an integral role in dismantling and degrading the skeletal muscle contractile apparatus.

The loss of skeletal muscle mass and strength associated with prolonged exposure to microgravity is a consequence of the decreased load on muscles during residence on the International Space Station (ISS), and is just one of several factors, including bone loss, redistribution of body fluids and immunosenescence^9^, that increases the risks associated with human deep space exploration and long-term habitation in lower Earth orbit. In mammals, postural (e.g, antigravity) muscles, such as soleus, are near maximally recruited under normal weight-bearing conditions^10^ and are thus the most severely affected by the absence of Earth’s gravity. Severe atrophy of these muscles is especially consequential in conditions where an astronaut’s return to terrestrial environments requires the ability to perform mission-related duties and even possibly respond to emergencies. Flight crew on the International Space Station currently allocate an average of 2.5 hours per day for exercise (including setup and breakdown of exercise equipment); however, even this is not sufficient to offset the effects of continuous exposure to microgravity on the musculoskeletal system^11^. Therefore, an adjunct therapy is necessary to better counter the effects of microgravity and permit continuity of mission-related duties in space and on the ground.

There have been prior examinations of muscle atrophy in low gravity conditions^12–14^, but these were conducted without the benefit of genetic tools to evaluate potential countermeasures to muscle loss. In the current study, MuRF1 knockout animals were compared to wild-type controls, to see if deletion of the MuRF1 E3 ubiquitin ligase would afford protection against low gravity-induced atrophy, as it has done in multiple models of muscle atrophy performed on Earth under normal Earth gravity conditions.

## Results

### MuRF1 Deletion Does Not Protect Against Microgravity-Induced Skeletal Muscle Loss

**Figure 1:**
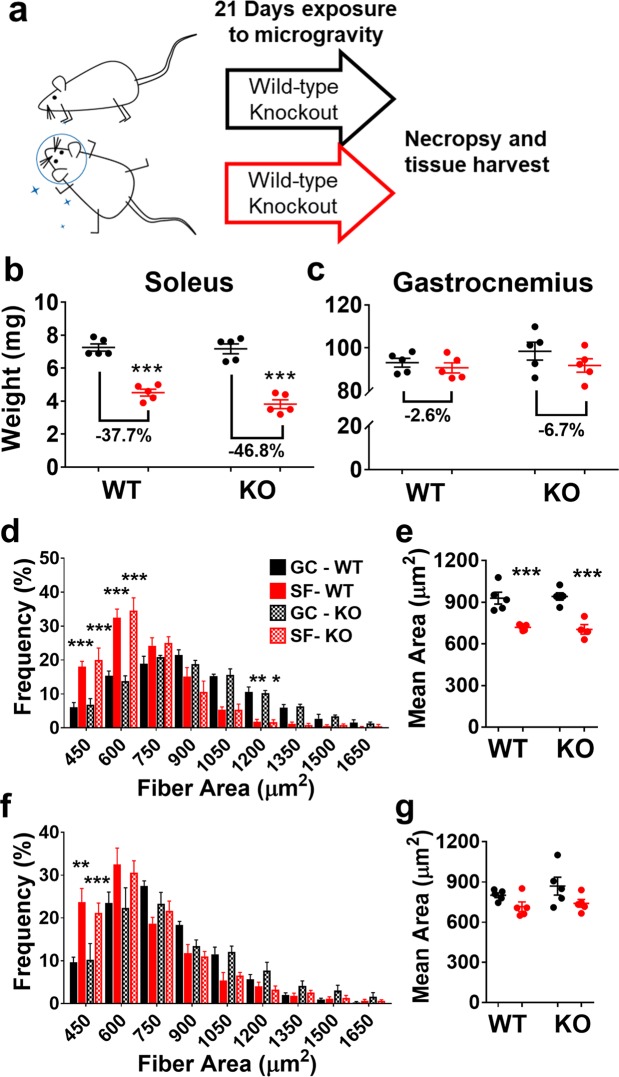
MuRF1 knockout does not protect against microgravity induced muscle loss. (**a**) Graphic representation of study design depicting WT and MuRF1 KO mice subjected to 21 days of Ground Control (black) or Spaceflight (red) conditions. (**b**) Both WT and MuRF1 KO mice present with significant soleus muscle loss with spaceflight. (**c**) Neither WT nor MuRF1 KO mice show significant muscle loss in the gastrocnemius muscle with spaceflight. Soleus type I fibers of both wild-type and MuRF1 KO mice show a leftward shift in fiber area frequency distribution (**d**) and a significant decline in (**e**) mean fiber area when subjected to microgravity. Type II fibers of both wild-type and MuRF1 KO mice show a moderate leftward shift in fiber area frequency distribution (**f**) but no significant difference in (**g**) mean fiber area. Data are presented as means ± SEM (n = 5/group). ***p < 0.001, Spaceflight vs. corresponding Ground Control by unpaired t-test for muscle weights. Data are presented as means ± SEM (n = 5/group). ***p < 0.001, **p < 0.01 and *p < 0.05, Spaceflight vs. corresponding Ground Control by one and two-way ANOVA with Tukey’s and Bonferonni’s post hoc for multiple comaprisons for fiber area frequency and mean fiber area, respectively. GC, Ground Control; SF, Spaceflight; WT, Wild-type; KO, Knockout.

Deletion of the E3 ubiquitin ligase MuRF1, which is responsible for the ubiquitination and proteasomal-mediated degradation of Myosin Heavy Chain^7^, amongst other substrates^8^, during skeletal muscle atrophy, was previously shown to spare skeletal muscle under multiple conditions that normally induce muscle atrophy^1^. All prior Earth-bound models had shown some degree of sparing of muscle mass in the setting of MuRF1 deletion^5,15–17^. We were therefore interested to know whether there would be enhanced maintenance of skeletal muscle in MuRF1 null animals in comparison to wild-type control mice after residence on the International Space Station (ISS), orbiting in a state of micro-gravity. Thirty-two week-old female wild type (WT) and MuRF1 knockout (KO) mice, on a C57BL6NTac background, were bodyweight-matched and randomized to Ground Control (GC) or Spaceflight (SF) for 21 days (Fig. 1a); baseline weights of all mice were recorded prior to launch (Supplemental Fig. 1). After 21 days of microgravity exposure on the ISS, Spaceflight (SF) mice were sacrificed by anesthetic overdose followed by cervical dislocation. Flight crew performed a gross dissection of the left hindlimb by cutting and removing the hindlimbs at mid-femur level and placing them in 10% neutral-buffered formalin. The remainder of the carcass was frozen and hindlimbs and carcasses were then stored in orbit until a more thorough dissection could be performed on the ground. Throughout the course of the experiment NASA veterinarians evaluated health status of the mice; ensuring their ability to maneuver around the habitat and adequately access food and water. A more detailed assessment of behavioral activity, including food intake, is published elsewhere and indicates that under microgravity conditions the mice in this experiment exhibited patterns of behavior that were comparable to their weight-bearing counterparts^18^. Ground Control (GC) mice were processed by the ground crew exactly as described for SF mice. Muscle weights were obtained from the formalin-fixed left hindlimbs from both GC and SF mice (Fig. 1b,c). The soleus muscle showed the largest degree of microgravity-induced atrophy in both the WT and KO mice (Fig. 1b; p < 0.001). This provided an early indication that micro-gravity induced muscle atrophy was distinct from most Earth-based models previously studied, because few models on Earth show such selectivity for the soleus muscle - though highlimb suspension^19,20^ and immobilization come closest^21^. Interestingly, MuRF1 KO mice lost 24% more soleus mass, albeit non-significantly, than WT controls (p = 0.0707), a trend that had never been observed with Earth-based models. Histological assessment of soleus (Supplemental Fig. 2a–h) and gastrocnemius (Supplemental Fig. 3a–h) of wild-type and KO mice showed no gross morphological changes in skeletal muscle subjected to microgravity conditions. Histomorphometric analysis of the soleus showed a significant leftward shift in type I fiber area frequency distribution (evident in both smaller and larger binned muscles; p < 0.05) and a significant decline in mean fiber area (p < 0.001; Fig. 1d,e). Type II fibers of wild-type and KO mice also showed a leftward shift in fiber area frequency distribution; however this was only significant in the smallest binned fibers (p < 0.01) so that mean fiber area was not significantly different (Fig. 1f,g). We observed a trend toward muscle loss in the gastrocnemius that was more prominent in the KO mice; however, this did not reach statistical significance (p = 0.2386; Fig. 1c). A major caveat to research on the ISS is a limitation in group sizes (in our case 5 mice per group) that is a result of limited laboratory space. It’s possible that a higher n and thus a more adequately powered study would have allowed for the ability to detect a significant difference in gastrocnemius muscle weights after three weeks of spaceflight. Overall, the lack of protection conferred by MuRF1 deficiency in microgravity is in contrast to what has previously been observed in MuRF1 knockout mice subjected to ground-based models of muscle atrophy.

### Transcriptional profiling reveals marginal differences in gene expression between Wild-Type and MuRF1 KO mice

**Figure 2:**
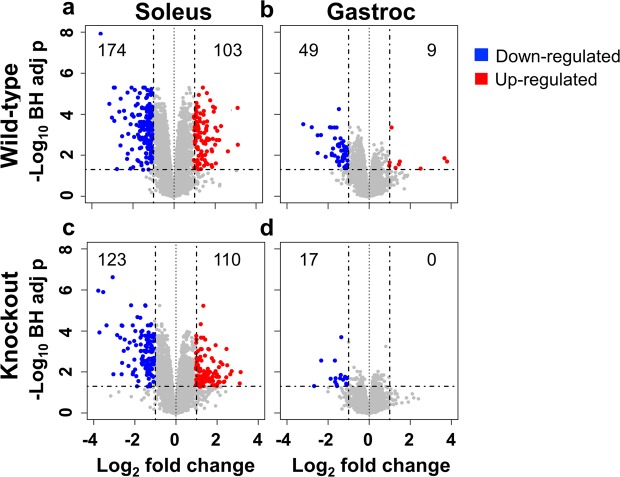
Volcano plots showing differentially regulated genes in soleus and gastrocnemius and wild-type and MuRF1 KO mice exposed to spaceflight or ground control conditions. (**a**) WT soleus shows more significant changes in gene expression than (**b**) WT gastrocnemius. MuRF1 KO is associated with less gene regulation in both (**c**) soleus and (**d**) gastrocnemius. The log_10_(BH adjusted p-values) were plotted against the log_2_(FC) in gene expression. Genes up-regulated by two-fold or more and with a BH corrected p-value < 0.05 are shown as red symbols, genes that were down-regulated by twofold or more and with a BH corrected p-value < 0.05 are shown with blue symbols. Gastroc, Gastrocnemius; BH, Benjamini–Hochberg; FC, fold-change.

In order to understand the mechanism by which skeletal muscles lose mass in space, we performed RNAseq analysis on the soleus and gastrocnemius to ascertain differences in gene expression profiles in mice subjected to microgravity; further WT and MuRF1 KOs were compared, to see if there were any differences between the two. As a quality control measure, we first evaluated MuRF1 levels and observed, as expected, no expression of MuRF1 (the *TRIM63* gene) in the KO animals. We also observed that MuRF1 transcripts were not up-regulated upon travel to space in soleus or gastrocnemius of WT animals (Supplemental Fig. 4a,b), another point distinguishing spaceflight from other inducers of muscle atrophy^5^. While this initial observation might not have been entirely surprising, since previous reports showed that MuRF1 expression is only up-regulated during the relatively acute stages of atrophy (i.e., <14 days), a subsequent experiment in WT mice demonstrated a lack of acute upregulation of MuRF1 in space as well (Supplemental Fig. 5); this lack of upregulation of MuRF1 on its own distinguishes spaceflight induced muscle atrophy from all Earth-based models studied, including hind-limb suspension. Consistent with the degree of muscle loss, the soleus showed a greater number of differentially regulated genes following 21 days of microgravity exposure (Fig. 2a–d). In the soleus, 103 and 110 genes were significantly up-regulated and 174 and 123 genes were significantly down-regulated in WT and KO mice, respectively (Fig. 2a,c; 2 fold change, adjusted p < 0.05). In contrast, the gastrocnemius showed only 9 and 0 genes to be significantly up-regulated and 49 and 17 genes significantly down-regulated in WT and KO mice, respectively (Fig. 2b,d; 2 fold change, adjusted p < 0.05). Due to the paucity of differentially regulated genes in the gastrocnemius, we henceforward focused exclusively on expression profiling of the soleus.

**Figure 3:**
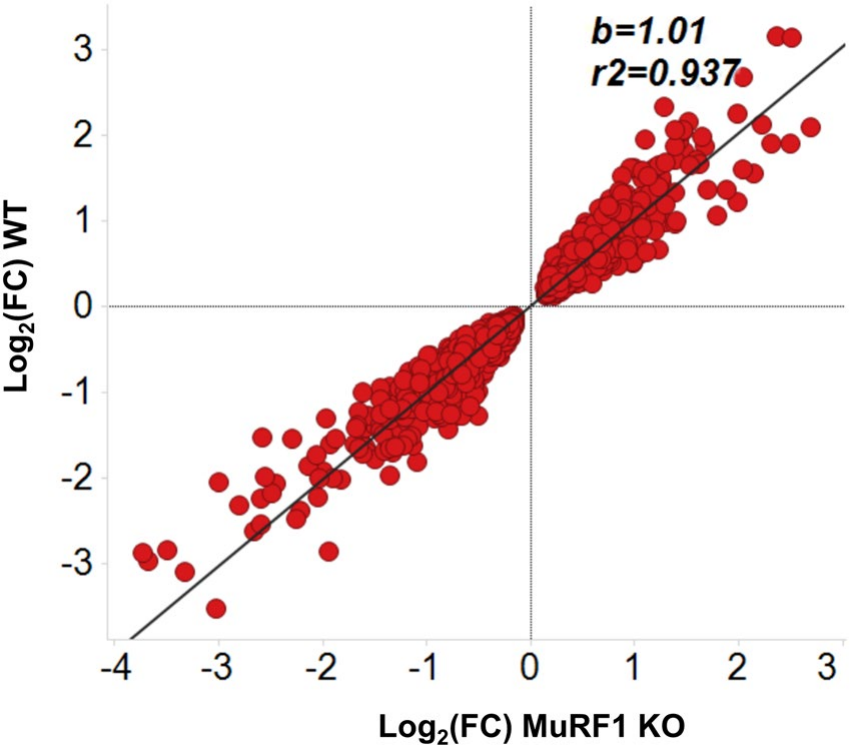
Correlation of soleus gene expression profiles of WT and MuRF1 KO mice exposed to microgravity. With a BH corrected p-value < 0.05 the log_2_(FC) of genes from WT mice were plotted against the log_2_(FC) of genes from MuRF1 KO mice. WT, Wild-type; KO, Knockout; BH, Benjamini–Hochberg; FC, fold-change.

We next interrogated transcriptome profiles of WT and MuRF1 KO mice to identify differences (or similarities) in individual genes affected by microgravity exposure. Using 2-fold change and <0.05 adjusted p value cutoff we observed a gene correlation with a slope of 1.01 and r^2^ of 0.937 (Fig. 3); indicating a large degree of similarity in gene expression profiles between WT and KO mice and corroborating the lack of efficacy observed in MuRF1 KO mice subjected to microgravity.

### MuRF1 KO Mitigates Muscle Loss Induced by Hindlimb Immobilization

**Figure 4:**
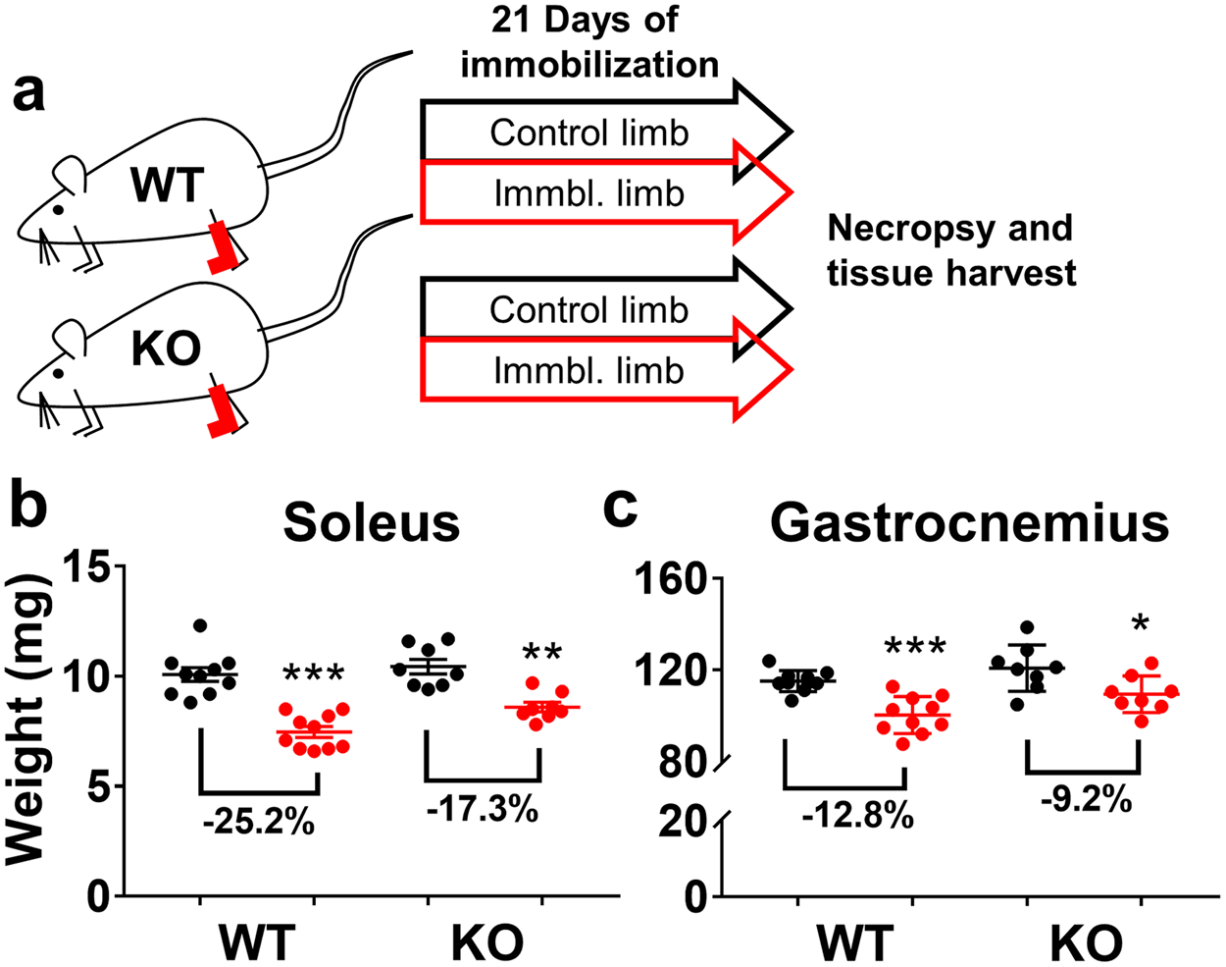
MURF1 deficiency mitigates muscle loss induced by hindlimb immobilization. (**a**) Graphic representation of study design depicting 21 days of unilateral hindlimb immobilization in WT (n = 10) and MuRF1 KO (n = 8) mice. Both WT and MuRF1 KO mice present with significant (**b**) soleus and (**c**) gastrocnemius muscle loss in the immobilized limb (red) relative to control limb (black). Muscle atrophy is mitigated in both muscles by MuRF1 KO. Data are presented as means ± SEM (n = 5/group). *p < 0.05, **p < 0.01, ***p < 0.001, immobilized vs. contralateral non-immobilized limb by paired t-test. WT, Wild-type; KO, Knockout.

Rodent hindlimb immobilization and hindlimb suspension are ground-based models that are often used to simulate the effects of microgravity exposure on the musculoskeletal system. In spaceflight, immobilization and hindlimb suspension, the soleus muscle presents with the greatest degree of mass loss; while exhibiting a shift in contractile properties and energy metabolism toward a faster and more glycolytic profile. Previous experiments have shown that muscles of MuRF1 KO mice are partially spared from atrophy when subjected to hindlimb suspension. Similarly, our next experiment evaluated MuRF1 KO mice subjected to hindlimb immobilization and was designed to match study duration and age and gender of mice that were on the ISS. Briefly, we immobilized the left hindlimbs of female C57BL/6NTac mice in a splint device, restricting movement in the ankle joint, for 21 days; the weight-bearing contralateral leg was used as a control (Fig. 4a). Similar to spaceflight, the soleus exhibited the greatest degree of muscle loss, with a significant decline of 25.2% and 17.3% in WT and KO mice (Fig. 4b; p < 0.001 and p < 0.01, respectively), indicating a 28% sparing of the muscle in the absence of MuRF1. The gastrocnemius (Fig. 4c) showed a significant decrease in weight in the WT (−12.8%; p < 0.01) but 33% less muscle loss in the KO (−9.2% atrophy; p < 0.05) mice; indicating a trend toward protection by MuRF1 KO in immobilization induced atrophy. This is in marked contrast to what was observed in mice subjected to microgravity for the same duration. Thus, while limb immobilization has classically been used as a standard model to simulate microgravity induced unloading, there are apparently some inherent characteristics of microgravity that cannot be adequately recapitulated on the ground.

### MuRF1 is not significantly up-regulated throughout a time course of microgravity induced atrophy

**Figure 5:**
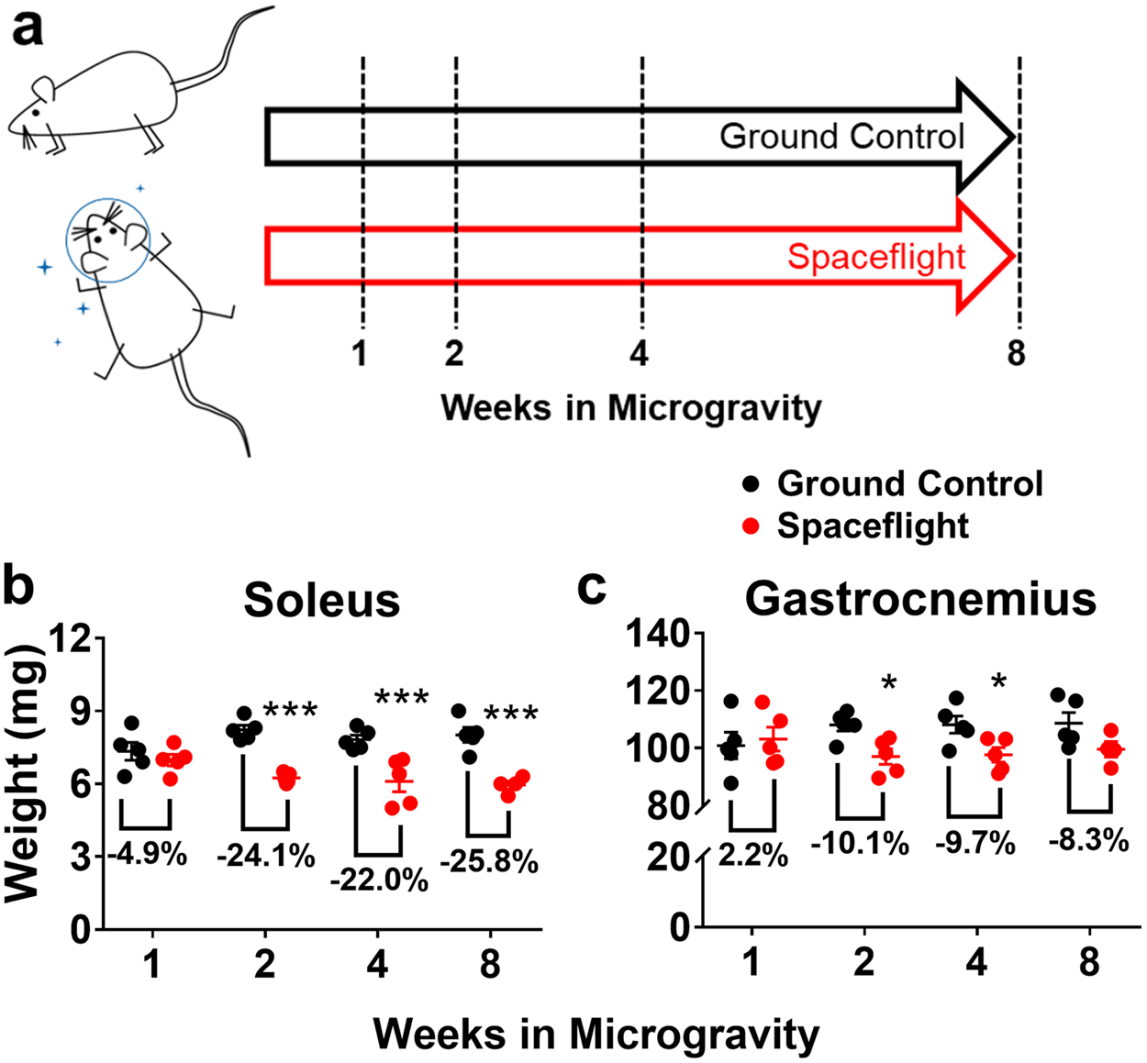
Time course of microgravity induced muscle atrophy. (**a**) Graphic representation of study design depicting mice subjected to 1, 2, 4 or 8 weeks of microgravity. (**b**) Mice present with significant soleus muscle loss after 2, 4 and 8 weeks of microgravity exposure. (**c**) Gastrocnemius shows marginal loss of mass after 2 and 4 weeks of microgravity only. Data are presented as means ± SEM (n = 4–5/group). *p < 0.05, ***p < 0.001, Spaceflight vs. corresponding Ground Control by unpaired t-test.

In order to better delineate skeletal muscle adaptations to microgravity we undertook a time course experiment that would allow for a more detailed assessment of both acute and chronic phases of microgravity induced atrophy. Wild-type female C57BL/6NTac mice were weight matched and randomized to GC or SF and then further randomized to an experimental duration of one, two, four or eight weeks (Fig. 5a). Both GC and SF mice were euthanized and necropsied as described earlier. Neither soleus nor gastrocnemius showed any significant atrophy after one week of microgravity. As with previous microgravity experiments, the soleus showed the greatest degree of weight loss, with a significant decrease of −24.1%, −22.0% and −25.8% at weeks two, four and eight, respectively (Fig. 5b; p < 0.001). The gastrocnemius presented with a moderate degree of atrophy at week two and four only (p < 0.01), with no significant difference detected at week 8 (Fig. 5c). Muscle loss in the gastrocnemius is in contrast to what was observed in our previous ISS experiment in MuRF1 KO mice (Fig. 1c) and may be due to inherent variability of the model, age of the mice and potential differences in age-associated behavior.

**Figure 6:**
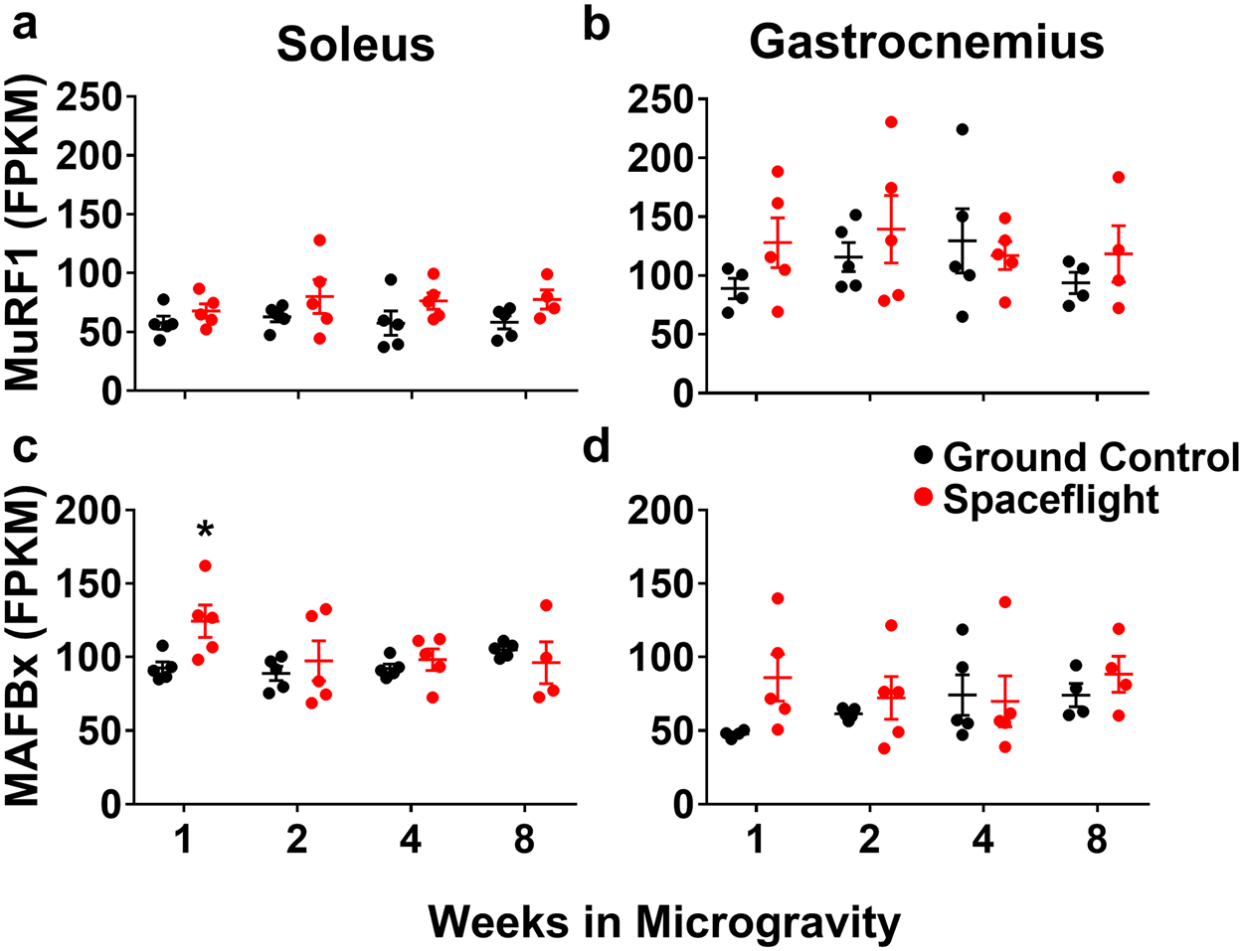
MuRF1 and MAFBx are not significantly up-regulated during a time course of microgravity exposure. Transcriptome profiling of the soleus (**a**,**c**) and gastrocnemius (**b**,**d**) showed that neither MuRF1 (**a**,**b**) nor MAFBx (**c**,**d**) were up-regulated with exposure to microgravity. Data are presented as mean FPKM values (n = 4–5/group). Unpaired t-test, Ground Control vs. Spaceflight. FPKM, fragments per kilo base per million mapped reads.

A global analysis of the gene expression profile showed that the expression of MuRF1 was largely unchanged in the SF group over the course of the experiment (Fig. 6a,b). An assessment of MAFBx (also called Atrogin-1), another prominent atrophy gene, showed an early and transient increase by microgravity in the soleus but not gastrocnemius (Fig. 6c,d). A more sensitive gene expression analysis of the gastrocnemius by qPCR corroborated our initial assessment of MuRF1 expression, showing no significant upregulation of MuRF1 over the course of the experiment (Supplemental Fig. 5a). In contrast, MAFBx expression, also assessed by qPCR, was significantly increased at week one (p < 0.05), but showed no further changes for the remaining duration of the experiment (Supplemental Fig. 5b). Taken together, the expression profiles of the two most well characterized atrophy genes corroborate the assessment that microgravity induced atrophy does not closely follow the classically delineated path toward muscle loss.

### A comparison of microgravity and hindlimb suspension shows regulation of genes that are distinct to ground and space

**Figure 7:**
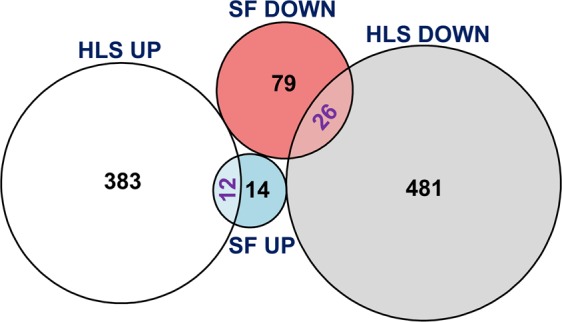
Euler diagram showing the up- and down-regulated genes altered by HLS and SF and their relationships. The area of each disjoint shape is proportional to the number of its elements as marked. SF, spaceflight; HLS, hindlimb suspension.

To further distinguish between muscle atrophy on the ground and that in space, we compared the soleus transcriptome from our time course experiment to a publicly available dataset from the soleus of mice subjected to 10 days of hindlimb suspension (HLS)^22^. Since the duration of the HLS experiment was 10 days, we selected time points from our microgravity time course that would provide the most temporally relevant comparison (i.e., one and two weeks). In addition to differences in the duration of experiments, the publicly available dataset was acquired from an analysis performed in male mice, in contrast to females that were housed on the ISS; therefore, data should be interpreted with this in mind. Our transcriptome analysis showed that a much larger proportion of genes were regulated by HLS (395 up-regulated and 507 down-regulated) than by SF (26 up-regulated and 105 down-regulated), with only a small proportion of regulated genes overlapping between the two models (12 up-regulated and 24 down-regulated, Hypergeometric test, p < 1e-16; Fig. 7). Pathway enrichment analysis (Table 1) was consistent with gene regulation in that more pathways were significantly enriched by HLS regulated genes (8 by down-regulated genes and 2 by up-regulated genes) and only two pathways were significantly enriched by SF – both enriched by down-regulated genes and with one pathway in common with HLS (Myogenesis pathway). The viral myocarditis pathway was the only significantly enriched pathway that was distinct to SF (Table 1); with associated genes falling in the myosin family (Myh2, Myh3, Myh10 and Myh7b). In regard to genes regulated by HLS, up-regulated genes were associated with collagen formation, extracellular matrix organization, estrogen responses and down-regulated were weakly enriched for circadian rhythm and KRAS signaling pathways (Table 1). The significantly smaller proportion of genes and associated pathways regulated by SF indicate unloading induced by microgravity is a more myogenic specific atrophy stimulus. Whereas, ground based models such as HLS, also activate ancillary and/or systemic responses that culminate in a more comprehensive induction of muscle loss; mechanisms that are more amenable to protection by MuRF1 deficiency.

**Table 1:** Pathway enrichment analysis with UP- or DOWN- regulated genes by HLS and SF.

	HLS		SF		Summary
	# Genes	Adj. P	# Genes	Adj. P	
**Pathways enriched by down-regulated genes**					
Hallmark myogenesis	18	**9.66E** − **04**	10	**1.62E** − **06**	Common
Reactome collagen formation	9	**2.99E** − **03**	2	8.35E − 01	HLS
Reactome extracellular matrix organization	9	**4.14E** − **02**	2	1.00E + 00	HLS
Hallmark estrogen response early	15	**2.46E** − **02**	2	1.00E + 00	HLS
Hallmark estrogen response late	20	**1.40E** − **04**	2	1.00E + 00	HLS
Kegg viral myocarditis	3	1.00E + 00	4	**4.52E** − **02**	SF
**Pathways enriched by up-regulated genes**					
Kegg circadian rhythm mammal	4	**4.24E** − **02**	0	1.00E + 00	HLS
Hallmark kras signaling dn	14	**1.84E** − **02**	0	1.00E + 00	HLS

## Discussion

Space travel holds great promise for humankind - the ability to explore and expand human knowledge beyond the confines of Earth, and to escape Earth if all else fails on this planet. But with that promise comes quite significant challenges, including the simple loss of gravity, which results in a range of deleterious effects on the body. Once free from gravity and the load it induces on skeletal muscle, that muscle responds in a manner that superficially resembles what happens when load is decreased on Earth - it atrophies, declining in mass, resulting in significant weakness when the gravitational load returns upon re-entry into the Earth’s atmosphere.

One way to determine if the atrophy induced by low gravity is similar to that on Earth is to use genetic tools that have been shown to be protective against atrophy on Earth. One such tool is the MuRF1 knockout mouse. MuRF1 is an E3 ubiquitin ligase which catalyzes the breakdown of skeletal muscle myosin heavy chain (MyHC)^7^, among other substrates^8^. MyHC is a major component of the contractile apparatus, and thus this breakdown causes loss of muscle mass and function, as a result of disuse or adaptation to decreased load on Earth. MuRF1 also mediates muscle breakdown in pathological settings, including the dramatic loss of muscle seen during cancer cachexia^23,24^, denervation^5^ and immobilization. In summary, MuRF1 KO mice - which have been genetically manipulated to be null for the MuRF1 protein - have demonstrated from 15 to 50% sparing of muscle mass, depending on model and muscle, in Earth bound models of atrophy^5,15–17^. Most importantly for discussions of microgravity-induced muscle atrophy, MuRF1 null mice have been used in the hindlimb suspension model, which has been advocated as a surrogate for space travel-induced atrophy, since it induces unloading of skeletal muscle, and the loss of MuRF1 has proven to be protective^5,17^. Therefore, we sought to determine if MuRF1 deletion would protect mice from skeletal muscle loss on the International Space Station, where they would experience low gravity conditions. As a positive control, a cohort of the same strain and gender were left on Earth, and subjected to hindlimb immobilization. Surprisingly, there was no skeletal muscle sparing of the MuRF1 knockouts in comparison to wild-type controls in space, whereas MuRF1 deletion continued to be effective in the Earth-based model of atrophy.

Furthermore, the animals on the ISS underwent a distinct pattern of atrophy, not seen in any of the Earth-bound models previously tested: it was predominantly the soleus muscle, which is mostly comprised of “slow oxidative” skeletal muscle fibers, that underwent atrophy in space, whereas in many models of atrophy on Earth, muscles that are rich in “fast glycolytic” fibers show a higher propensity for atrophy^25^. In settings of hindlimb suspension and immobilization, soleus is also lost, but loss of other muscles is not far behind.

A molecular analysis of the soleus muscle (albeit, between males on the ground and females in space) demonstrated that skeletal muscle atrophy following spaceflight, and thus under low gravity conditions, proceeds in a unique manner in comparison to other forms of atrophy. Indeed, a much smaller proportion of genes were regulated in space, in comparison to an Earth-bound hindlimb suspension model, giving some demonstration that the space model is unique. Most surprisingly, expression of the MuRF1 gene itself does not increase in space, whereas it does during hindlimb suspension. This simple difference could be sufficient to explain why the deletion of the gene is not sparing. As to the genes that were regulated under low gravity conditions, they’re consistent with genes that are involved in muscle differentiation, suggesting that some of the signaling mechanisms inducing differentiation are hampered in low-gravity conditions. Interestingly, muscle-differentiation genes were also seen to be inhibited by treatment with myostatin, a protein that inhibits muscle growth, in a prior study^26^.

While it is clearly more convenient to use Earth-bound models as surrogates for spaceflight, it seems clear from this study that in order to understand the molecular changes that occur as a result of escape from Earth’s gravity, animals and human need to be studied in space. Neither mouse nor human evolved in low gravity, and thus the changes induced by spaceflight are difficult to predict, and can therefore best be understood from a result of study and experimentation in space. This study clearly demonstrated the utility of work on the International Space Station, as a laboratory to understand the effects of low gravity.

## Materials and Methods

All methods were performed in accordance with the relevant guidelines and regulations.

### MuRF1 KO mice on the International Space Station (ISS)

All procedures were approved by Ames Research Center, Kennedy Space Center and Novartis Institutional Animal Care and Use Committees prior to conducting experiments.

Ten female muscle RING finger protein 1 (MuRF1) KO mice were weight-matched and randomized to spaceflight (SF; n = 5) or ground control (GC; n = 5) and 10 female wild-type litter mates randomized to SF (n = 5) or GC (n = 5), both on C57BL/6NTac background and 32 weeks of age at launch, were obtained from Taconic Biosciences (Rensselaer, NY). Approximately four weeks prior to launch (L-28), mice were received at Kennedy Space Center (KSC) and maintained in standard vivarium housing on a 12:12 hour dark/light cycle. Mice were provided NASA Type 12 Nutrient-upgraded Rodent Food Bars and supplied with deionized, autoclaved water, ad libitum. At L-21 mice were introduced to wire floors for preconditioning to the interior surfaces of the habitats aboard the International Space Station (ISS). At L-10 mice from each genotype were randomized to SF or GC groups and at L-2 loaded into transporters at double density and at L-1 loaded into the Dragon SpaceX Capsule. On L +4, mice were transferred into habitats aboard the ISS. Ground control mice were subjected to the same conditions as SF mice, with the exception of launch and spaceflight but including (on a 4 day offset) temperature, humidity, and CO_2_ partial pressure. In addition mice were evaluated daily by a NASA veterinarian via video link to the ground. Video link was established on L +5 and also included assessment of animal behavior, including food intake and activity levels. Detailed evaluation of behavior is published elsewhere^18^.

Twenty-one days post-launch mice were euthanized by flight crew aboard the ISS by intraperitoneal injection of a 0.4 ml overdose of Euthasol diluted in sterile saline, followed by cervical dislocation to confirm euthanasia. Flight crew performed a gross dissection of the left hind limb by cutting and removing at mid-femur level and placing in 10% neutral-buffered formalin. The hind limb was stored in formalin for approximately 24 hours, then transferred to 70% ethanol. The remainder of the carcass was frozen and hind limbs and carcasses were then stored in orbit until a more thorough dissection could be performed on the ground. Mice were slow frozen in a mini cold bag (MCB) containing three ice bricks frozen to −130 °C. Five mice were necropsied each day and tissues and carcasses were placed in the MCB. The MCB temperature was −140 °C at the time of placing the first mouse and −80 °C at the time of placing the last mouse. After necropsy and freezing of the fifth mouse tissues and carcasses were then transferred to long term storage at −130 °C. Ground Control mice were processed by the ground crew exactly as described for SF mice.

### MuRF1 KO mice and hindlimb immobilization

All procedures were approved by Novartis Institutional Animal Care and Use Committee prior to conducting experiments. Mice were socially housed with environmental enrichment in a temperature (22 °C) and humidity-controlled (43%) room. Mice were maintained on a 12-h light-dark cycle and provided food (5053, PicoLab Rodent Diet 20; LabDiets) and water ad libitum. Fifty-two week old female MuRF1 KO (n = 8) and wild-type littermates (n = 10) on C57BL/6NTac background were subjected to unilateral hindlimb immobilization. Briefly, mice were anesthetized by inhalation of 2–3% isoflurane in oxygen and left hindlimb was immobilized in a splint device, restricting movement in the ankle and maintaining the joint at the neutral position; the weight-bearing contralateral leg was used as a control. Animals were monitored on a daily basis for abrasions or edema of the immobilized limb and to ensure ambulation and access to food and water. After 21 days animals were sacrificed by CO_2_ asphyxiation and muscles collected and wet weights recorded.

### Atrophy time course experiment on the ISS

All procedures were approved by Ames Research Center, Kennedy Space Center and Novartis Institutional Animal Care and Use Committees prior to conducting experiments. Forty female wild-type mice on C57BL/6NTac background were obtained from Taconic Biosciences (Rensselaer, NY) and were 16 weeks of age at the time of launch. Pre and post-flight time lines and procedures were performed exactly as described for the MuRF1 KO experiments; differences are described below. Mice were weight matched into two groups and randomized to SF or GC, then further randomized into a one, two, four or eight week time course group (n = 5/group). One animal in the eight-week SF group was noted by flight crew to present with poor clinical condition and was thus ordered by the NASA veterinarian to be euthanized early; all remaining animals were sacrificed on schedule. Following anesthetic overdose and cervical dislocation both hind limbs were removed by disarticulation at the hip joint. The left hind limb was immediately placed in 4% paraformaldehyde for approximatey 48 hours, rinsed for ten minutes in PBS and transferred to fresh PBS containing 0.02% sodium azide. The right hind limb was placed into a 5 ml cryotube and frozen, as was the remainder of the carcass. Ground Control mice were processed by the ground crew exactly as described for SF mice.

### Histology

Formalin-fixed muscles were bisected and paraffin-embedded, and 10-μm cross sections were taken at the midbelly region. Sections were deparaffinized, rehydrated and subjected to Masson’s trichrome stain. In regard to immunohistochemitry, deparaffinized and rehydrated10-μm cross sections were subjected to antigen retrieval by heating in Rodent Decloaker solution (BioCare Medical, Pacheco, CA) at 80 °C for 30 min. Sections were blocked in 5% goat serum in PBS and incubated in rabbit anti-laminin (Sigma) diluted in 1:100 in blocking solution. Sections were then incubated in goat anti-rabbit secondary antibody conjugated to Alexa 488 (Life Technologies) diluted 1:200 in blocking solution. For detection of type II fibers sections were further incubated in anti-fast (type II) MHC conjugated to alkaline phosphatase (Sigma, St. Louis, MO) diluted 1:50 in PBS for 60 min at room temperature. Whole stained sections were scanned on an Olympus VS120 fluorescent slide scanner.

### RNA extraction and quantitative PCR

Immediately after dissection muscles were lysed in 1 ml of TRIzol reagent (Thermo Fisher Scientific, 15596018) by using Lysing Matrix A tubes (MP Biomedicals, 116910050; Lucerna Chem AG, Lucerne, Switzerland). Total RNA isolation was carried out using TRIzol reagent according to the manufacturer’s instructions and a TURBO DNA-free™ Kit (Thermo Fisher Scientific, AM1907) was used for complete digestion of DNA. 500 ng of RNA was reverse transcribed to cDNA using the High-Capacity RNA-to-cDNA™ Kit (Thermo Fisher Scientific, 4387406). Quantitative PCR (qPCR) was performed by using 10 ng of cDNA per each sample and TaqMan® Universal PCR Master Mix (Thermo Fisher Scientific, 4324018). Gene-specific primers with FAM-labeled probes were from Thermo Fisher Scientific: Mafbx, Mm00499518_m1; Murf1, Mm01185221_m1. Tbp, Mm01277042_m1; Hprt, Mm01545399_m1; and Rplp0, Mm00725448_s1 were used as internal controls. PCR cycling conditions on software supplied with ViiA™ 7 System (Applied Biosystems) were as follows: 50 °C for 2 min, 95 °C for 10 min, 40 cycles at 95 °C for 15 s, and 60 °C for 1 min. Data were expressed as Ct values and used for the relative quantification of targets with the ΔΔCt calculation to give N-fold differences. Data were transformed through the equation 2^−ΔΔCt^.

### RNAseq library preparation and sequencing

RNA was prepared as described for quantitative qPCR. The concentration of RNA was measured by fluorometric quantitation using the Quant-iT RNA Assay Kit, broad range (ThermoFisher Scientific, Illkirch, France) on a FLx800 microplate reader (BioTek Instruments, Sursee, Switzerland). Total RNA was processed using the TruSeq Stranded mRNA or TruSeq RNA Library Preparation Kit v2 library preparation kits (Illumina, San Diego, CA, USA) according to the manufacturer’s instructions. Wherever possible, we used 250–400 ng of total RNA and enriched adapter-ligated fragments with 12 cycles of PCR. For the microglia extracts, we used between 7 and 35 ng of input, and 15 cycles of PCR. Library fragment size distribution was determined using a D1000 ScreenTape on an Agilent 2100 Bioanalyzer Instrument (Agilent Technologies, Waldbronn, Germany) and quantified on a Qubit 3.0 Fluorometer using Qubit dsDNA High sensitivity or Broad range reagents (ThermoFisher Scientific, Illkirch, France). Up to 12 libraries were pooled and loaded on 1–2 lanes of either an Illumina HiSeq. 2500 (16.5 pM final loading concentration) or HiSeq. 4000 (210 pM) using HiSeq v4 or HiSeq. 4000 PE cluster kits and reagents, respectively (Illumina, San Diego, CA, USA). For each library, we generated on average 45 million paired-end, 76 base pair reads.

### RNAseq data processing and analysis

Sequencing reads were aligned to the GRCm38/mm10 (https://www.ncbi.nlm.nih.gov/assembly/GCF_000001635.26) mouse reference transcriptome using STAR 2.5.2b with quantMode GeneCounts enabled. Quality metrics on read duplication, transcript integrity, splice junction saturation and gene body coverage were checked using the RSeQC package, version 2.6.2. Differential expression analysis was performed with edgeR and limma R/Bioconductor (https://www.r-project.org/, https://www.bioconductor.org/) packages after filtering for genes with count per million (cpm) values above 1 in at least 3 samples. Genes were identified as differentially expressed based on 2 criteria: absolute fold change (FC) ≥2 and Benjamini and Hochberg correction p-value ≤ 0.05. Plots were generated using R gplots and TIBCO Spotfire.

### Pathway enrichment analysis

Pathway enrichment analysis was implemented on differentially expressed genes derived from HLS and SF, with the criteria described earlier. The annotated gene sets, including canonical pathways and hallmark pathways from Molecular Signatures Database (http://software.broadinstitute.org/gsea/msigdb/), were used in the analysis. For each pathway, the corresponding gene set were converted to mouse homologs using the R package msigdbr. The pathway was then compared with the up- and down-regulated gene list from HLS and SF, followed by a hypergeometric test to measure the statistical significance, e.g. p-value, of the over-representation.

### Statistical analysis

Number of animals and experiments per group (n), and statistical tests are reported in the figure legends. A p-value of ≤ 0.05 was considered statistically significant and a BH-corrected p-value of ≤ 0.05 was considered statistically significant for RNAseq and pathway analysees. Unless otherwise specified, data were analyzed by GraphPad Prism 6 software (San Diego, CA).

## Supplementary information

Supplementary Data
