# Supplementary material for: Skeletal muscle in MuRF1 null mice is not spared in low-gravity conditions, indicating atrophy proceeds by unique mechanisms in space
Source: Sci Rep. 2019 Jun 28;9:9397. doi: 10.1038/s41598-019-45821-9 (PMC6599046; doi:10.1038/s41598-019-45821-9)
Supplement: Supplementary file 1 — Supplementary Data [file 41598_2019_45821_MOESM1_ESM.pdf]

Skeletal muscle in MuRF1 null mice is not spared in low-gravity conditions

Samuel M. Cadena[1], Yunyu Zhang[1], Jian Fang[1], Sophie Brachat[2], Pia Kuss[2], Elisa Giorgetti[2], Louis Stodieck[3], Michaela Kneissel[2], David J. Glass[1]*

[1]Novartis Institutes for Biomedical Research, 181 Massachusetts Avenue, Cambridge, MA 02139, USA

[2]Novartis Institutes for Biomedical Research, Forum 1, Novartis Campus, 4056 Basel, Switzerland

[3]BioServe Space Technologies, Department of Aerospace Engineering Sciences, University of Colorado, Boulder, CO 80302 USA

*to whom correspondence should be addressed: david.glass@novartis.com

key words: muscle atrophy, MuRF1, outer-space, space flight, skeletal muscle, gene expression

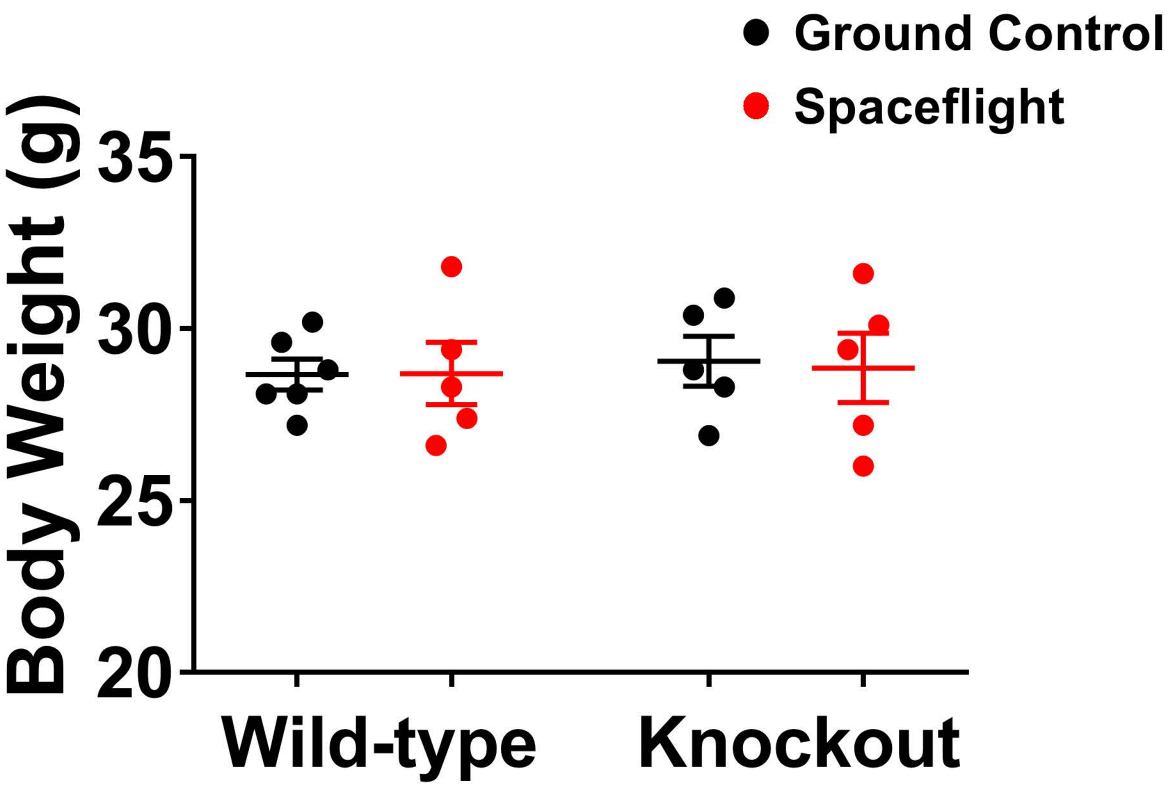

Supplemental Figure 1

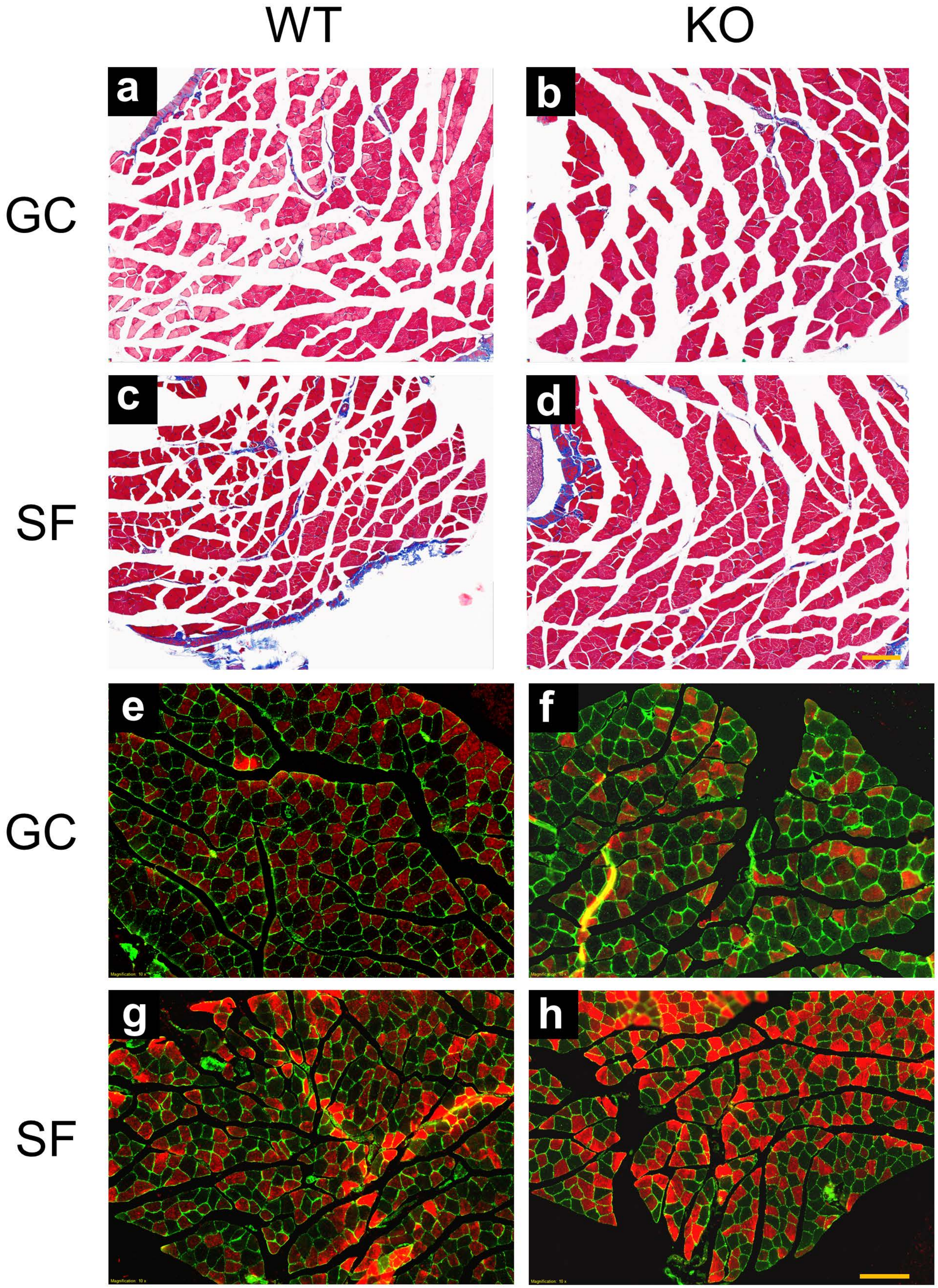

Supplemental Figure 2

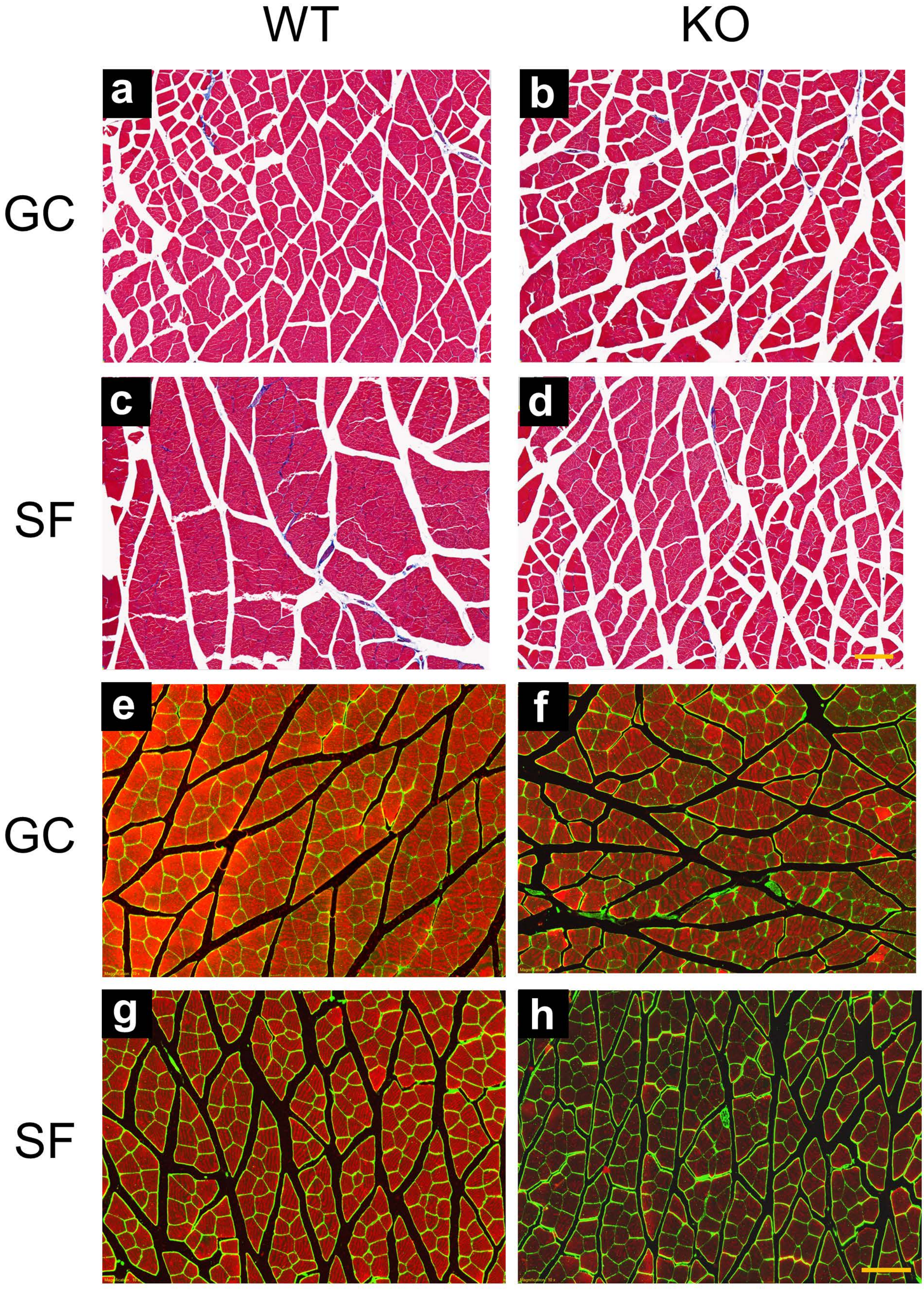

Supplemental Figure 3

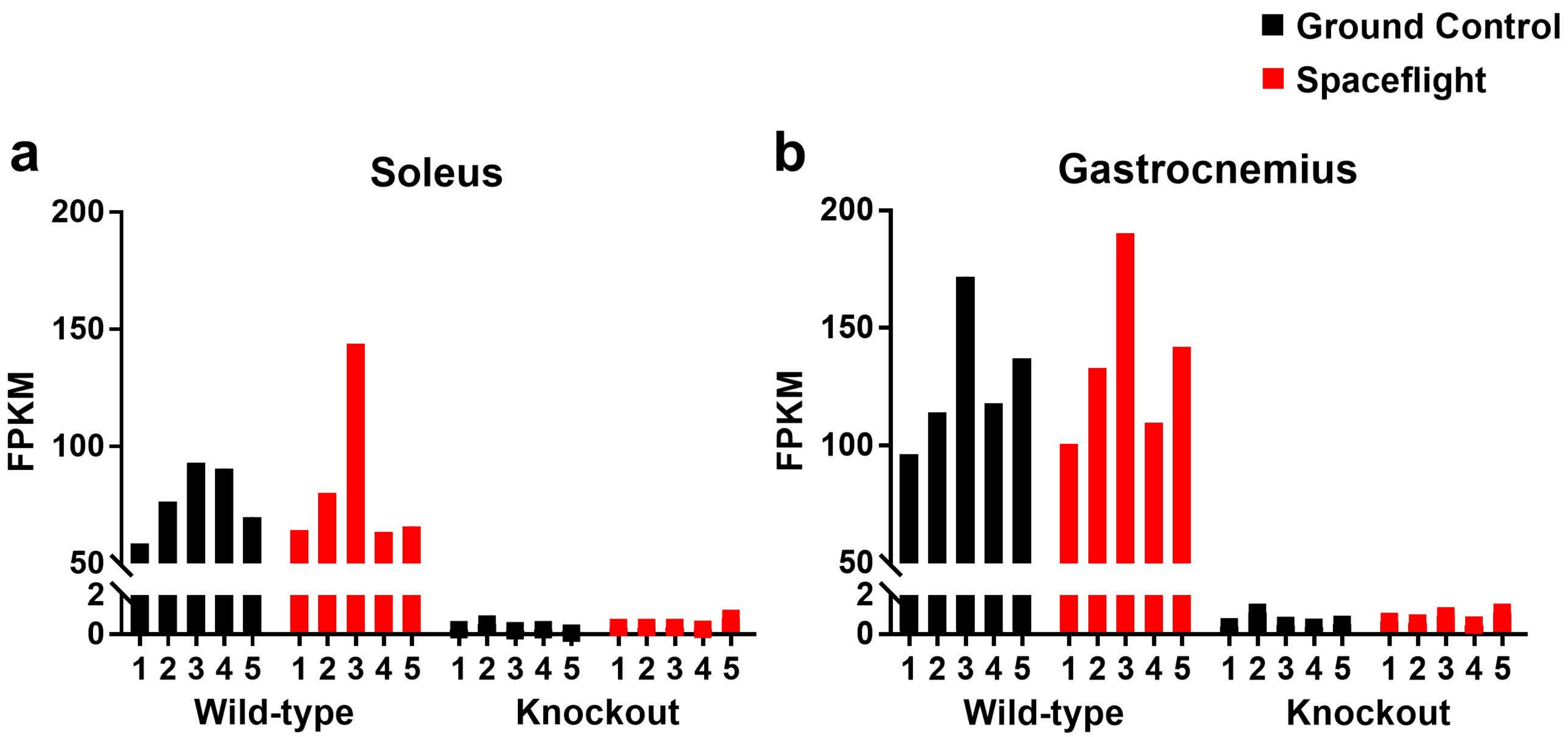

Supplemental Figure 4

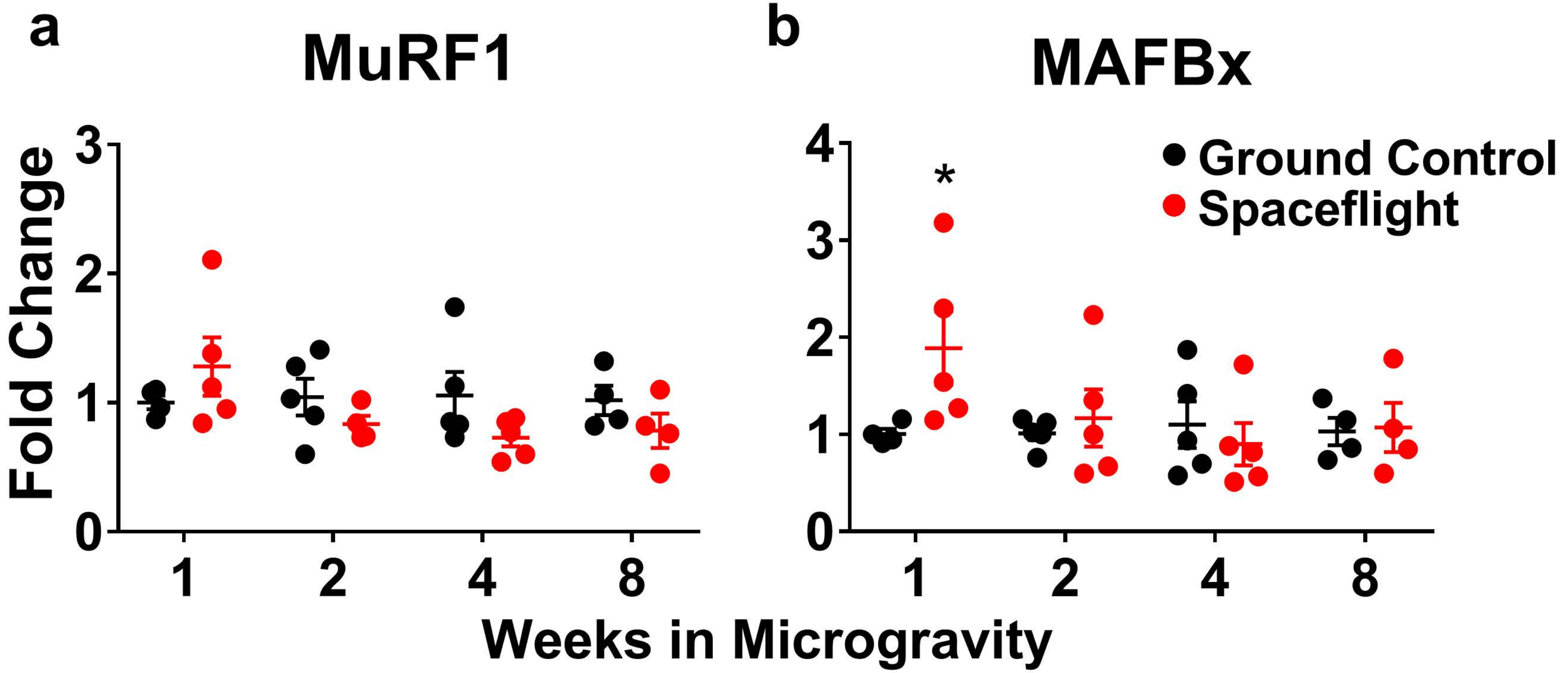

Supplemental Figure 5

SUPPLEMENTAL FIGURE LEGENDS

Supplementary Figure 1

Pre-flight body weights of Wild-type and MuRF1 KO mice. No significant difference ($p>0.05$) between pre-flight body weights (g) of female Wild-type and MuRF1 KO mice or Ground Control and Spaceflight groups (n=5/group). Data presented as means ± SEM. One-way ANOVA.

Supplemental Figure 2

Histological assessment of soleus muscle. Representative images of Masson's trichrome stained soleus muscles cross-sections (**a-d**) and immunohistochemistry with anti-laminin antibody (green) and anti-fast (type II) myosin heavy chain antibody (red; **e-h**).

Supplemental Figure 3

Histological assessment of gastrocnemius muscle. Representative images of Masson's trichrome stained gastrocnemius muscles cross-sections (**a-d**) and immunohistochemistry with anti-laminin antibody (green) and anti-fast (type II) myosin heavy chain antibody (red; **e-h**).

Supplemental Figure 4

MuRF1 expression in Wild-type and MuRF1 KO mice following 21 days of microgravity exposure. MuRF1 expression was not detected in MuRF1 KO mice and MuRF1 was not up-regulated with spaceflight in WT mice in (**a**) soleus or (**b**) gastrocnemius muscles. Data are presented as individual FPKM values (n=5/group). $p>0.05$, Ground Control vs. Spaceflight by unpaired t-test. WT, Wild-type; KO, Knockout; FPKM, fragments per kilo base per million mapped reads.

Supplemental Figure 5

qPCR validation of MuRF1 and MAFBx gene expression. MAFBx but not MuRF1 is moderately and transiently up-regulated with exposure to microgravity. The gastrocnemius muscle of mice exposed to a time course of microgravity showed (**a**) no change in MuRF1 expression but (**b**) a marginal significant upregulation of MAFBx at one week only. Data are presented as means ± SEM (n=4-5/group). *$p<0.05$, Spaceflight vs. corresponding Ground Control by unpaired t-test.
